# Angiotensin-(1–7) inhibits inflammation and oxidative stress to relieve lung injury induced by chronic intermittent hypoxia in rats

**DOI:** 10.1590/1414-431X20165431

**Published:** 2016-09-01

**Authors:** W. Lu, J. Kang, K. Hu, S. Tang, X. Zhou, S. Yu, Y. Li, L. Xu

**Affiliations:** Division of Respiratory Disease, Renmin Hospital of Wuhan University, Wuhan, China

**Keywords:** Ang-(1–7), Lung injury, Obstructive sleep apnea, Inflammation, Oxidative stress, Chronic intermittent hypoxia

## Abstract

Obstructive sleep apnea is associated with inflammation and oxidative stress in lung tissues and can lead to metabolic abnormalities. We investigated the effects of angiotensin1–7 [Ang-(1–7)] on lung injury in rats induced by chronic intermittent hypoxia (CIH). We randomly assigned 32 male Sprague-Dawley rats (180–200 g) to normoxia control (NC), CIH-untreated (uCIH), Ang-(1–7)-treated normoxia control (N-A), and Ang-(1–7)-treated CIH (CIH-A) groups. Oxidative stress biomarkers were measured in lung tissues, and expression of NADPH oxidase 4 (Nox4) and Nox subunits (p22phox, and p47phox) was determined by Western blot and reverse transcription-polymerase chain reaction. Pulmonary pathological changes were more evident in the uCIH group than in the other groups. Enzyme-linked immunosorbent assays and immunohistochemical staining showed that inflammatory factor concentrations in serum and lung tissues in the uCIH group were significantly higher than those in the NC and N-A groups. Expression of inflammatory factors was significantly higher in the CIH-A group than in the NC and N-A groups, but was lower than in the uCIH group (P<0.01). Oxidative stress was markedly higher in the uCIH group than in the NC and N-A groups. Expression of Nox4 and its subunits was also increased in the uCIH group. These changes were attenuated upon Ang-(1–7) treatment. In summary, treatment with Ang-(1-7) reversed signs of CIH-induced lung injury via inhibition of inflammation and oxidative stress.

## Introduction

Obstructive sleep apnea (OSA) is a disorder that leads to metabolic abnormalities. The condition is characterized by obstruction of the upper airways with unconscious repetitive pauses in breathing during sleep, causing daytime sleepiness and other problems. It is usually associated with a reduction in blood oxygen saturation. Hypoxia can lead to epithelial and endothelial cell injury in lung tissues. OSA affects at least 2–4% of the adult population, largely older adults, and its prevalence has been reported to exceed 30% in those aged 65 years and above (1). Mechanisms involved in OSA pathogenesis include oxidative stress, systemic inflammation, and endothelial dysfunction.

The lungs are among the most oxygenated organs in the human body. Apnea causes acute physiological changes, including alveolar hypoventilation and pulmonary artery vasoconstriction. OSA-related high-frequency intermittent hypoxia (IH) is characterized by cyclical changes in hypoxemia with reoxygenation, similar to ischemia-reperfusion injury, and contributes to increased production of reactive oxygen species (ROS), which is distinctly different from sustained low-frequency hypoxia (2). In addition, hypoxia can cause tissue edema, inflammatory cell infiltration, elevated levels of cytokines, and oxidative stress. Proinflammatory cytokines interleukin-6 (IL-6), tumor necrosis factor (TNF-α), and IL-8 play an important role in promoting lung injury. IL-6 is produced by various immune and non-immune cells, including vascular endothelial cells, monocytes/macrophages, keratinocytes, and fibroblasts (3). IL-8 functions include chemotaxis and activation of neutrophils, as well as chemotaxis of basophils, T lymphocytes, and other inflammatory cells (4). Decreased antioxidant capacity and increased free radical levels have been implicated in the pathological changes associated with pulmonary diseases (5). Superoxide dismutase (SOD) is a critical antioxidant that can effectively eliminate superoxide anions and protect cells from oxidative damage. Malondialdehyde (MDA) is a stable metabolite of lipid peroxidation. MDA, SOD, catalase (CAT), and Nox4 are established markers used to evaluate oxidative stress. Previous studies (6–8) have found that IH inhibits lymphocyte and neutrophil apoptosis in lung tissues via the effects of a range of factors including proinflammatory cytokines; additionally, hypoxia promotes increased ROS production and release of active proinflammatory mediators, which contribute to systemic inflammation, endothelial dysfunction, and subsequent cardiovascular disease development in OSA. Thus, IH contributes to diverse multiorgan chronic morbidity and mortality. The CIH model in rats, in which hypoxia-reoxygenation episodes are stimulated and several cardiovascular pathological features of OSA are reproduced, is the gold standard model for studying the mechanisms involved in OSA pathogenesis.

Proof of the efficacy of angiotensin-(1–7) [Ang-(1–7)] as a novel therapeutic agent for many diseases has been revealed in preclinical studies. Angiotensin-converting enzyme 2 (ACE-2) degrades Ang II by removal of a single amino acid to generate the heptapeptide Ang-(1–7). The lung tissues of both mice and rats treated with bleomycin displayed significantly reduced levels of ACE-2 mRNA, protein, and enzymatic activity, indicating that ACE-2 is protective against experimental lung injury (9). Ang-(1–7) is a counter-regulatory mediator of Ang II, which also appears to be protective against cardiovascular disease (10). A few studies have explored the use of Ang-(1–7) administration in lung injury in various animal models and demonstrated some efficacy (11–13). However, the underlying mechanism remains unclear and further studies are needed to elucidate the specific protective pathways involved. In the present study, we investigated inflammation and oxidative stress in lung tissues using a rat hypoxia model to explore the effects of Ang-(1–7) in lung injury induced by chronic intermittent hypoxia (CIH). We tested the hypothesis that Ang-(1–7) mitigates lung injury induced by CIH and exerts a protective effect via inhibition of inflammation and oxidative stress.

## Material and Methods

### Experimental protocols

Male Sprague-Dawley rats (n=32; body weight, 180–200 g) were purchased from the Experimental Animal Center of Wuhan University (Wuhan, China). Eight rats (exposed to normoxia control, NC group) were randomly selected to establish baseline levels for all biomarkers used in the study. Embedded with an osmotic mini-pump (Alzet 2004; Alza, USA), the remaining 24 rats were randomly subdivided into three groups, denoted as uCIH (CIH exposure without any treatment), CIH-A (CIH exposure + Ang-(1–7) infusion at 400 ng·kg^–1^·min^–1^ for 28 days), or N-A (normoxia exposure + Ang-(1–7) infusion at 400 ng·kg^–1^·min^–1^ for 28 days). This study was approved by the Ethics Committee of Wuhan University and conducted in accordance with the Declaration of Helsinki and the Guide for the Care and Use of Laboratory Animals, as adopted and promulgated by the United National Institutes of Health. Rats were housed in departmental animal chambers, with a 12:12 h light-dark cycle under standard laboratory conditions (temperature: 25±2°C; humidity: 60±5%). Rats were provided standard rodent chow and allowed free access to water. At the end of the experiment, all rats were sacrificed. Every effort was made to minimize the number of rats used and their suffering during the experiments.

The CIH model was established according to previously published methods (14), Briefly, sealed chambers were used to generate a hypoxic environment. Next, pure nitrogen and compressed air were distributed into each chamber through timed solenoid valves. Using 90-s cycles, pure nitrogen was infused into each chamber for the first 30 s until the minimum oxygen concentration reached 5%. Compressed air was infused for the remaining 60 s to allow the oxygen concentration in the chambers to gradually return to 20.9%. For the NC and N-A group, air was forced into the chamber. For all groups, exposure experiments were performed between 8:00 am and 4:00 pm.

### Collection and storage of blood and tissue samples

Rats from each experimental group were sacrificed by exsanguination via intracardiac puncture. Blood samples were subjected to centrifugation and sera were stored at –80°C. Lung tissues were harvested and fixed in 4% phosphate-buffered formaldehyde for histopathological analyses. Renal tissues were immediately frozen in liquid nitrogen and stored at –80°C.

### Histological staining

Lung tissue specimens were fixed with 10% formalin, embedded in paraffin, cut into 5-μm thick sections, and mounted onto slides. Sections were then stained with hematoxylin and eosin (H&E). Light microscopy was performed using a Nikon E400 light microscope (Nikon Instrument Group, USA).

### Immunohistochemical analysis

Lung sections were incubated twice in xylene for 5 min, followed by dehydration in a gradient of histology-grade ethanol for 5 min, then rehydrated and rinsed in phosphate-buffered saline (PBS). Slides were then incubated in 30% H_2_O_2_ for 10 min at room temperature and washed twice with distilled water and PBS. Bovine serum albumin was added to the slides for 15 min. Next, excess serum was removed and rabbit anti-rat IL-6 and TNF-α antibodies (1:200; Boster, China) as well as anti-rat IL-8 (1:2000; Abcam, UK) were added, followed by incubation for 1 h at room temperature. After three washes with PBS for 3 min each, secondary antibodies (goat anti-rabbit IgG-HRP, 1:50,000; Boster) were added and incubated for 15 min at room temperature, followed by three 3-min washes with PBS. Histological analyses were performed using a Nikon E400 light microscope (Nikon Instrument Group). Thirty randomly chosen lung tissue sections were analyzed using Image Pro Plus (version 6.0; Media Cybernetics, USA) at a magnification×400, and integrated optical density (IOD) was used as a relative standard amount of positive staining.

### ELISA assay

Blood samples were centrifuged at 377.3 *g* for 10 min and stored at –80°C until further analysis. Plasma levels of TNF-α, IL-6, IL-8 and MCP-1 were determined using solid-phase sandwich enzyme-linked immunosorbent assay (ELISA) kits specific for these factors, and absorbance was measured at 450 nm using a plate reader (BioTek ELx800, USA).

### Detection of MDA level and antioxidant enzyme activity

Frozen lung specimens were homogenized in tissue lysis buffer (Beyotime, China). After lysis for 15 min on ice, homogenates were centrifuged at 377.3 *g* for 15 min at 4°C. MDA content in the supernatants were measured using commercially available kits (Jiancheng Bioengineering Institute, China). Briefly, MDA levels in the homogenates were determined spectrophotometrically by measuring the presence of thiobarbituric acid-reactive substances. Phosphoric acid (1%, 3 mL) and 1 mL of a 0.6% thiobarbituric acid solution were added to 0.5 mL of plasma that had been pipetted into a tube. The mixture was then heated in boiling water for 45 min. After the mixture had cooled, the color was extracted into 4 mL of n-butanol. The absorbance was measured in an enzyme-labeled instrument at 532 nm. The amount of lipid peroxide was calculated to represent the amount of thiobarbituric acid-reactive substances, which are products of lipid peroxidation. Results are reported in nmol/mg of protein according to a standard graph prepared from measurements of standard solutions of 1,1,3,3-tetramethoxypropane. SOD activity in renal tissues was measured using a commercial assay kit (Jiancheng Bioengineering Institute) following the manufacturer's instructions. This assay kit employs a thiazole salt for the detection of superoxide anions by producing a colored product. The absorbance was measured at a wavelength of 450 nm. One unit of SOD was defined as the total amount of enzyme needed to produce 50% dismutation of superoxide radicals. To assay CAT, 100 μL of lung homogenate was diluted to a total volume of 1.2 mL with sodium phosphate buffer (50 mM, pH 7.0) and mixed with 1 mL of 30 mM H_2_O_2_ solution. The OD of each sample was measured at 240 nm for 3 min against a reagent blank-containing buffer rather than lung homogenate. CAT values are reported as the absorbance at 405 nm. Enzyme activity units were defined according to the degradation of 1 μmol of H_2_O_2_·s^–1^·mg^–1^ of protein. The enzyme activity are reported as units/mg of protein.

### Real-time RT-PCR

Total RNA was extracted from tissue using Trizol reagent (Invitrogen, USA). Reverse transcription was performed to produce cDNA from total RNA with oligo (dT), and then the fragments were amplified using a SYBR Green-based assay kit (Invitrogen) according to the manufacturer's instructions. Thermal cycling conditions comprised an initial denaturation step at 94°C for 10 min, followed by 40 cycles of 94°C for 30 s, 60°C for 30 s, and 72°C for 60 s. *GAPDH* was used for normalization, and data were analyzed using the 2^-ΔΔCt^ method. Primer sequences were as follows: *Nox4*, forward: 5′-GCCACTTGATCCCTTGCTG-3′, reverse: 5′-AACGACCTCGCAATGACATC-3′. *p22phox*, forward: 5′-GCTGCCTATCGGGATGGTGAA-3′, reverse: 5′-ATGGACGCCACGATCACGAA-3′. *p47phox*, forward: 5′-GCGATGCTGCCTACTTGTGA-3′, reverse: 5′-TGAGGTTGCTGCCACAGAGA-3′. *GAPDH*, forward: 5′-TCAACGGCACAGTCAAGG-3′, reverse: 5′-TGAGCCTTCCACGATG-3′.

### Western blotting

Lung tissues were used for western blot analysis. Protein concentrations were measured using the bicinchoninic acid (BCA) protein assay (Thermo Scientific, USA). Equal amounts of boiled protein in the loading buffer were separated via NuPAGE 10% Bis-Tris SDS-PAGE (Life Technologies, China) and then electrophoretically transferred to polyvinylidene fluoride membranes (Millipore, USA). Membranes were subsequently incubated with primary antibodies (rabbit anti-Nox4, 1:300, Elabscience, China; rabbit anti-p22phox and -p47phox, 1:300, Santa Cruz Biotechnology, Inc., USA), washed three times with TBST buffer, and incubated at room temperature for 1 h in the presence of a horseradish peroxidase-conjugated secondary antibody (goat anti-rabbit IgG, 1:50,000; Boster). After the blots were washed three times with TTBS buffer, they were developed and exposed using enhanced chemiluminescence on Hyperfilm X-ray films. The resultant protein bands were quantified by densitometry (QuantityOne 4.5.0 software; Bio-Rad Inc., USA).

### Statistical analysis

Data are reported as means±SE. Statistical analysis was performed using SPSS v17.0 software (IBM, USA). Statistical comparisons between groups were conducted using a one-way analysis of variance (ANOVA) test, and an LSD test was performed for multiple comparisons. P<0.05 indicated a significant difference.

## Results

### Histopathological characteristics

**Figure 1:**
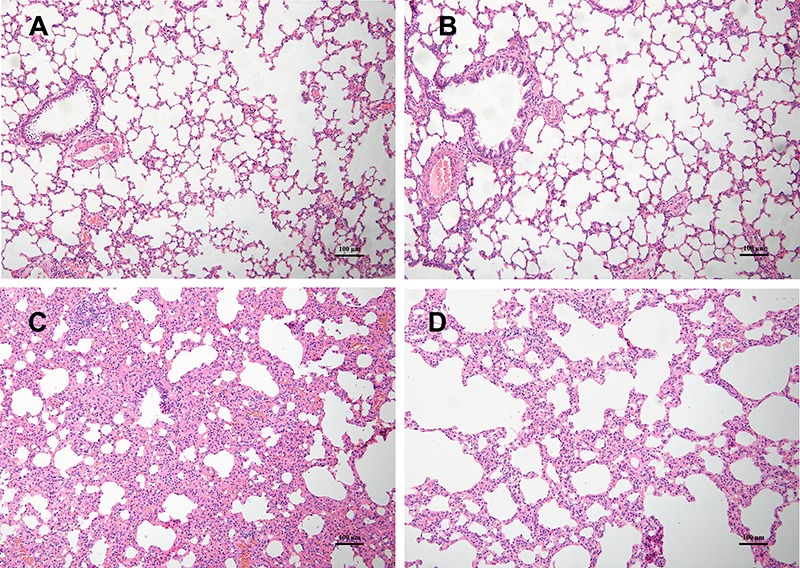
Histopathological changes in lung tissue samples of the four groups. Hematoxylin and eosin stain (×100 magnification, bar 100 μm). *A* and *B*, NC and N-A groups (n=8): normal lung structure. *C*, uCIH group (n=8): increased alveolar wall thickness, edema, bleeding, and infiltration of inflammatory cells. *D*, CIH-A group (n=8): mild structural destruction and inflammatory infiltration. NC: normoxia control group; N-A: NC and Ang-(1–7) supplemented group; uCIH: untreated chronic intermittent hypoxia group; CIH-A: CIH and Ang-(1–7) supplemented group

H&E staining revealed that there were almost no histological differences between the NC and N-A groups. However, uCIH group showed increased alveolar wall thickness, edema, bleeding, and infiltration of inflammatory cells compared with controls. CIH-A group showed less inflammation and distortion of pulmonary architecture compared with those in uCIH group (Figure 1).

### Ang-(1–7) downregulated the release of proinflammatory cytokines in the lungs

**Figure 2:**
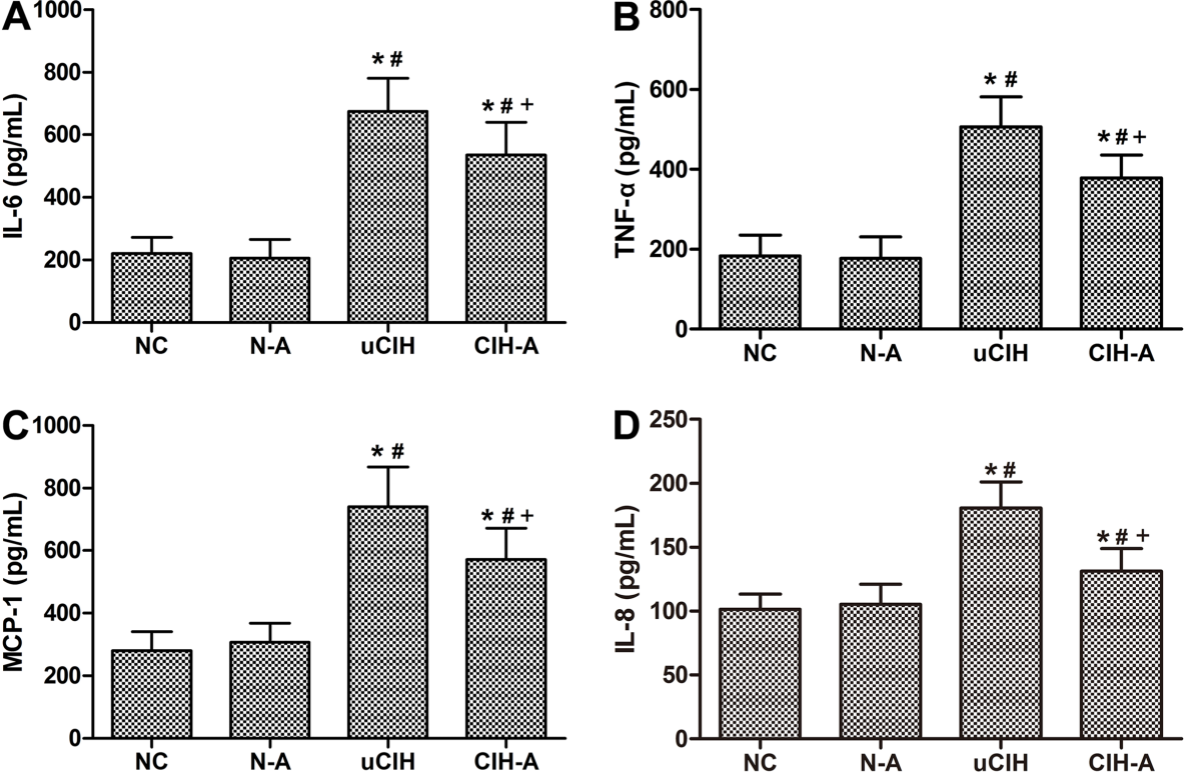
Changes in the levels of proinflammatory cytokines (ELISA): *A*, Interleukin (IL-6); *B*, tumor necrosis factor (TNF-α); *C*, monocyte chemotactic protein 1 (MCP-1), and *D*, interleukin (IL-8). Data are reported as means±SE. NC: normoxia control group (n=8); N-A: NC and Ang-(1–7) supplement group (n=8); uCIH: untreated chronic intermittent hypoxia group (n=8); CIH-A: CIH and Ang-(1–7) supplement group (n=8). *P<0.05 *vs* NC; ^#^P<0.05 *vs* N-A; ^+^P<0.05 *vs* uCIH (ANOVA).

**Figure 3:**
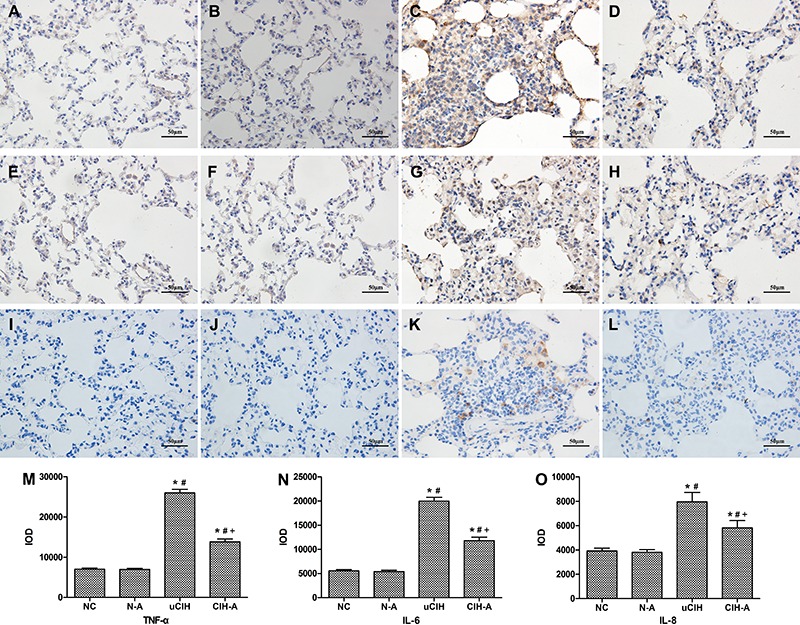
Immunohistochemical expression of TNF-α and IL-6 in rat lungs. *A*-*D*, TNF-α; *E*-*H*, IL-6; *I*-*L*, IL-8. Representative photomicrographs of lung immunohistochemical analysis (400×, bar 50 μm). Little expression could be seen in alveolar epithelial cells, and inflammatory cells in *A*, *E*, *I* (NC group) and *B*, *F*, *J* (N-A group). A strong positive expression occurred in *C*, *J*, *K* (uCIH group) and staining intensity was attenuated in *D*, *H*, *L* (CIH-A group). *M–O*, comparison of integrated optical density (IOD) values among the 4 groups: *M*, TNF-α; *N*, IL-6; *O*, IL-8. Data are reported as means±SE. NC: normoxia control group (n=8); N-A: NC and Ang-(1–7) supplemented group (n*=*8); uCIH: untreated chronic intermittent hypoxia group (n*=*8); CIH-A: CIH and Ang-(1–7) supplemented group (n=8). *P<0.05 *vs* NC; ^#^P<0.05 *vs* N-A; ^+^P<0.05 *vs* uCIH (ANOVA).

In the uCIH group, ELISA revealed that the levels of serum TNF-α, IL-6, IL-8 and MCP-1 increased sharply after CIH administration compared with those in the NC and N-A groups (all P<0.05). In contrast, CIH-A group had significantly lower levels of TNF-α, IL-6, IL-8 and MCP-1 compared with those of the uCIH group (uCIH *vs* CIH-A: TNF-α: 506.59±74.75 *vs* 378.55±57.15 pg/mL, IL-6: 675.25±106.29 *vs* 535.00±100.82 pg/mL, IL-8: 180.68±20.39 *vs* 131.05±17.87 pg/mL, MCP-1: 740.25±126.99 *vs* 571.33±100.82, all P<0.05; Figure 2). Immunohistochemical analysis showed a significant increase in TNF-α, IL-6, and IL-8 expression in the lung tissues of the uCIH group compared to that in the CIH-A group (uCIH *vs* CIH-A: TNF-α: 25,979.23±907.38 *vs* 13,827.20±712.59; IL-6: 19,979.23±811.61 *vs* 11,827.20±703.88; IL-8: 7,958.01±771.15 *vs* 5,810.09±604.93, all P<0.05). No statistical difference in the expression of these cytokines was observed between the NC and N-A groups (P>0.05; Figure 3).

### Ang-(1–7) attenuated CIH-induced oxidative stress in the lungs

**Figure 4:**
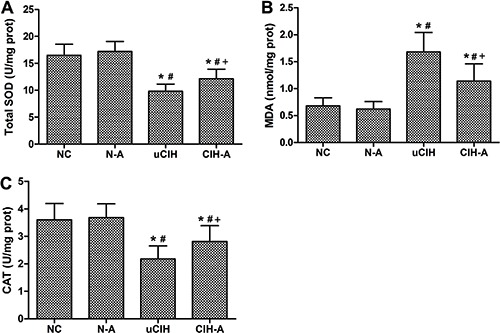
Ang-(1–7) reduced oxidative stress in lung tissues. *A*, Total SOD activity; *B*, MDA content; *C*, CAT level. Results are reported as means±SE (n=8 rats per group). NC: normoxia control group; N-A: NC and Ang-(1–7) supplemented group; uCIH: untreated chronic intermittent hypoxia group; CIH-A: CIH and Ang-(1–7) supplemented group. *P<0.05 *vs* NC; ^#^P<0.05 *vs* N-A;^+^P<0.05 *vs* uCIH (ANOVA).

Enhanced oxidative stress was observed in uCIH animals, as evidenced by elevated MDA levels in their lung tissues (P<0.05). Moreover, similar changes were observed for lung SOD activity and CAT levels, which were both significantly decreased in uCIH rats (P<0.05). In contrast, in CIH-A group, treatment with Ang-(1–7) effectively enhanced SOD activity and CAT levels and decreased MDA content in lung tissue (P<0.05). There were no significant differences in the levels of these three oxidative stress indices between the NC and N-A groups (P>0.05; Figure 4).

### Ang-(1–7) suppressed CIH-induced expression of Nox4 and its subunits in the lungs

**Figure 5:**
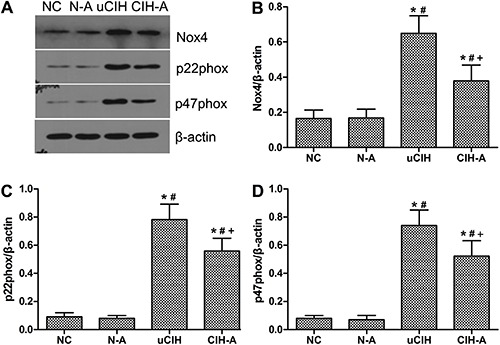
Western blot analysis of protein levels in rat lung tissue. *A*, Concentrations of Nox4, p22phox and p47phox were determined by western blot analysis; *B*, Nox4/β-actin, *C*, p22phox/β-actin; *D*, p47phox/β-actin. Results of a representative experiment are shown. Ang-(1–7) down-regulated the CIH-induced elevation of Nox4, p22phox and p47phox expression. Data are reported as means±SE. NC: normoxia control group (n=8); N-A: NC and Ang-(1–7) supplemented group (n=8); uCIH: untreated chronic intermittent hypoxia group (n=8); CIH-A: CIH and Ang-(1–7) supplemented group (n=8). *P<0.05 *vs* NC; P<0.05 *vs* N-A; ^+^P<0.05 *vs* uCIH (ANOVA).

**Figure 6:**
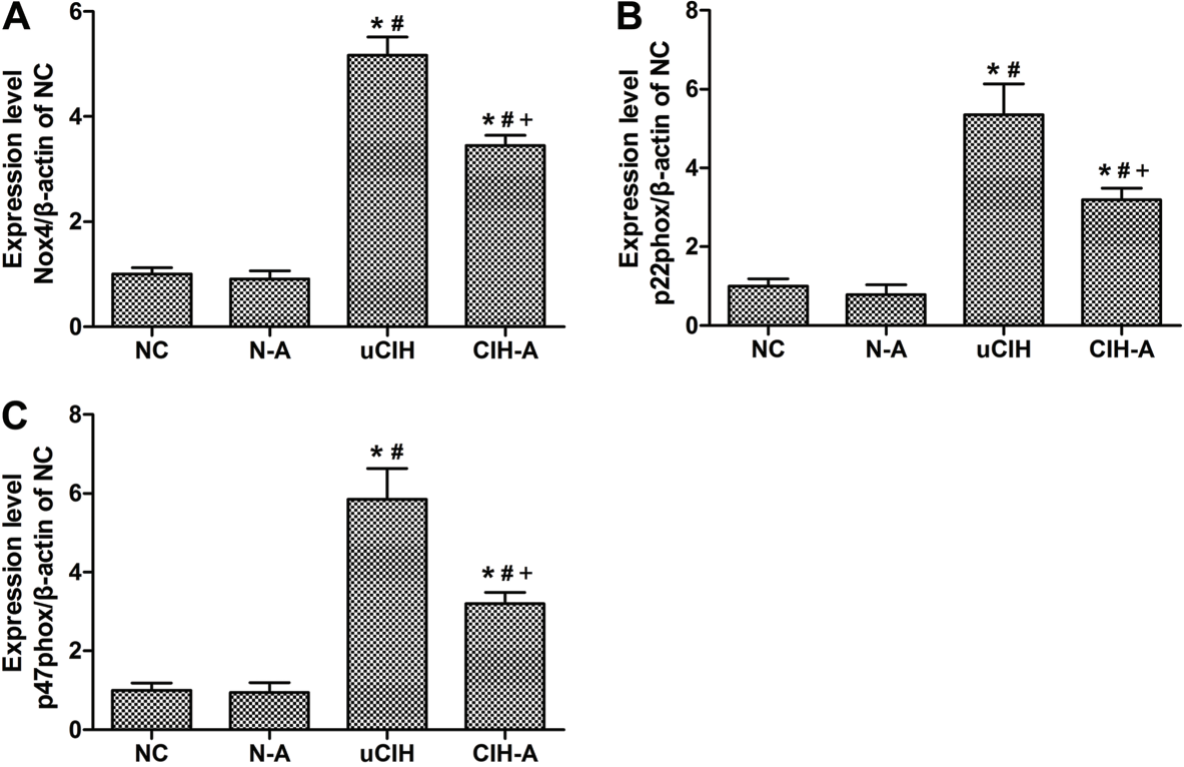
Gene expression of Nox4, p22phox and p47phox analyzed by real-time PCR. Amplification of β-actin served as a control, and the levels were compared with normoxia control (NC) lungs taken as 100% (n=8). N-A: NC and Ang-(1–7) supplemented group; uCIH: untreated chronic intermittent hypoxia group; CIH-A: CIH and Ang-(1–7) supplemented group. *P<0.05 *vs* NC; ^#^P<0.05 *vs* N-A; ^+^P<0.05 *vs* uCIH (ANOVA).

Protein expression of Nox4 and its subunits was markedly higher in the uCIH and CIH-A rats compared with that in both the NC and N-A groups (all P<0.05). Moreover, Nox4, and p22phox and p47phox subunit levels were significantly higher in uCIH rats than in CIH-A rats (uCIH *vs* CIH-A: Nox4: 0.501±0.10 *vs* 0.319±0.09; p22phox: 0.782±0.11 *vs* 0.559±0.09; p47phox: 0.740±0.11 *vs* 0.522±0.08, P<0.05). These findings indicate that Ang-(1–7) mitigated the CIH-induced increase in Nox4 expression, consistent with the results of the western blot analysis (Figure 5). Additionally, real-time PCR was performed to confirm the protein expression results, and a similar expression pattern was observed (Figure 6).

## Discussion

We studied CIH-induced lung injury in rats and investigated the protective effect of Ang-(1–7). The lung inflammatory response is regulated by the coordinated functions of cytokines, chemokines, and adhesion molecules. Rassler et al. (15) reported that continuous hypoxia promotes lung edema, inflammatory cell infiltration, and thickening of the alveolar interval, and that hypoxia extension gradually aggravates lung tissue inflammation. Induction of cytokines such as TNF-α and IL-6 is involved in transcriptional reprogramming induced by CIH. TNF-α levels are elevated in OSA patients compared with controls. Madjdpour et al. (16) showed that decreased alveolar oxygen concentration leads to lung tissue inflammation and increased expression of TNF-α in the alveolar lavage fluid of rats. TNF-α levels were increased from 54 pg/mL to 89 pg/mL (68% increase, P<0.01) upon exposure of rat alveolar macrophage cells to hypoxia for 5 h *in vitro*. Furthermore, Minoguchi et al. (17) reported that TNF-α modulates physiological sleep, and that the level of TNF-α is correlated with the severity of OSA. Ryan et al. (18) found that circulating TNF-α levels were higher in OSA patients (2.56 pg/mL; IQR, 2.01 to 3.42 pg/mL) than in control subjects (1.25 pg/mL; IQR, 0.94 to 1.87 pg/mL; P<0.001). IL-6 is another inflammatory cytokine that may play an important role in inducing chemokine and leukocyte recruitment (19–21). The levels of IL-6 and its receptors are elevated in OSA patients compared with that of controls. Oyama et al. (22) reported that the basal apnea-hypopnea index (AHI) correlates with TNF-α levels (r=0.420, P=0.017), and that plasma concentrations of TNF-α and IL-6 decreased significantly with continuous positive airway pressure therapy. In OSA patients, the levels of IL-6 were increased compared with those in control subjects and were proportional to AHI severity. IL-8 has strong chemotactic properties and activates neutrophil chemotaxis, as well as chemotaxis of basophilic granulocytes, T lymphocytes, and other inflammatory cells involved in angiogenesis. IL-8 has been shown to dramatically increase after lung ischemia-reperfusion injury (23). In the current study, we found that the concentrations of IL-6, TNF-α and IL-8 in the sera and lung tissues of uCIH rats were significantly increased compared with those in the NC and N-A rats. Increased *in vitro* secretion of both MCP-1 and TNF-α derived from alveolar macrophages of rats exposed to 5% O_2_ for 5 h has also been previously demonstrated (16). These findings indicate that CIH leads to increased inflammation, consistent with our results.

Accumulating evidence suggests that Nox is a major source of ROS, most notably of •O_2_^–^. Under physiological conditions, a certain amount of intracellular •O_2_^–^ is required for normal redox homeostasis in tissues (24). However, excessive •O_2_^–^ in the endothelium stimulates vasoconstriction and inflammation, which mutually reinforce each other, resulting in endothelial dysfunction (25). It has also been recognized that reduced Nox levels play a critical role in generating ROS, and drugs that block Nox activity may be potential therapeutic agents for reducing oxidative stress. Nisbet et al. (26) reported an increase in the expression of Nox4 and p22phox in lung homogenates of rats in a CIH protocol that simulated the desaturation of oxygen observed in patients with OSA (O_2_ from 21 to 10% every 90 s, during 8 weeks). In addition, hypoxia leads to an upregulation of Nox4, p22phox and p47phox mRNA in homogenized lung tissue in C57BL/6N mice (27). In the current study, the levels of SOD and CAT in rat lung tissues were decreased in the NC group relative to the uCIH group, and the levels of Nox4 and its subunits, p22phox and p47phox, as well as those of MDA, were significantly increased.

The discovery of Ang-(1–7) by Santos et al. (28) shed new light on angiotensin metabolism and the regulation of the renin-angiotensin-aldosterone system (RAAS). The heptapeptide Ang-(1–7) is predominantly formed by ACE2-mediated cleavage of the octapeptide Ang II. The ACE2/Ang-(1–7) axis has emerged as a key determinant of lung injury. In both pulmonary and non-pulmonary systems, Ang-(1–7) has been shown to counteract the detrimental effects of Ang II through the Mas receptor. Ang-(1–7) has also been shown to inhibit Ang II-induced signaling through activation of a phosphatase and apoptosis (29). Several studies have demonstrated the therapeutic potential of Ang-(1–7) as highly protective against lung inflammation (12,30,31). Magalhães et al. (32) studied the anti-inflammatory action of Ang-(1–7) in a model of chronic lung inflammation. Activation of Ang-(1–7) was also shown to modulate the expression of proinflammatory cytokines in a model of pulmonary hypertension, wherein decreased expression of TNF-α and IL-6 was observed (33,34). Papinska et al. (35) reported that treatment with Ang-(1–7) reduced the extent of oxidative stress and inflammation in lung and increased the overall area that participates in oxygen-carbon dioxide exchange. Another study demonstrated that infusion of Ang-(1–7) attenuated inflammation and pathological changes, such as increased alveolar septal thickening and cellular infiltration, in all studied time-windows in acute lung injury (30). Jawien et al. (36) reported that relative p22phox expression was significantly decreased in AVE (Ang-(1–7) receptor agonist)-treated mice, whereas it tended to increase in A-779 (Ang-(1–7) receptor antagonist)-treated mice, as compared with controls. This suggests that Ang-(1–7) inhibits Nox expression, which is crucial for the oxidative stress induced by CIH. Ang-(1–7) could mitigate the inflammation and oxidative stress induced by CIH by counteracting Ang II. However, the half-life of Ang-(1–7) in plasma is relatively short, and it may therefore have limited therapeutic effectiveness. In our study, implantation of osmotic mini-pumps ensured continuous delivery of Ang-(1–7), thus overcoming this disadvantage. The bioavailability of Ang-(1–7) after subcutaneous injection has been reported to be 98%, suggesting that almost all of the injected Ang-(1–7) entered the bloodstream, which makes this route of delivery highly effective. We found that the thickening of the alveolar septa and inflammatory cell infiltration associated with lung injury could be partially reversed by treatment with Ang-(1–7). When compared with the uCIH group, concentrations of IL-6, TNF-α and IL-8 as well as the oxidative stress levels, decreased in the CIH-A group after treatment with Ang-(1–7). These findings are consistent with those of the aforementioned studies. Taken together, the experimental evidence provided here indicates a protective role for Ang-(1–7) against oxidative stress and inflammation in lung injury induced by CIH.

In conclusion, we demonstrated a protective role of Ang-(1–7) against CIH-induced lung injury in rats, which was partially mediated by inhibition of proinflammatory cytokine release and oxidative stress.
